# The effect of adjuvant therapies on long-term outcome for primary resected synovial sarcoma in a series of mainly children and adolescents

**DOI:** 10.1007/s00432-021-03614-6

**Published:** 2021-07-17

**Authors:** Monika Scheer, Christian Vokuhl, Sebastian Bauer, Jörg Fuchs, Steffan Loff, Beate Timmermann, Marc Münter, Anton George Henssen, Bernarda Kazanowska, Felix Niggli, Ruth Ladenstein, Gustaf Ljungman, Ewa Koscielniak, Thomas Klingebiel, the European Cooperative Weichteilsarkom Studiengruppe [CWS]

**Affiliations:** 1 Department of Pediatric Hematology and Oncology, corporate member of Freie Universität Berlin and Humboldt Universität zu Berlin, Charité–Universitätsmedizin Berlin, Augustenburger Platz 1, 13353 Berlin, Germany; 2 Pediatric Pathology, University Hospital of Bonn, Bonn, Germany; 3 Sarcoma Center, West German Cancer Center, University of Duisburg‐Essen, Essen, Germany; 4 Department of Pediatric Surgery and Urology, University Children’s Hospital, Tübingen, Germany; 5 Pediatric Surgery, Klinikum Stuttgart, Olgahospital, Stuttgart, Germany; 6 Department of Particle Therapy, West German Proton Therapy Centre Essen (WPE), West German Cancer Centre (WTZ), West German, University Hospital Essen, Essen, Germany; 7 Department of Radiation Oncology, Klinikum Stuttgart, Stuttgart, Germany; 8 Department of Pediatric Oncology, University of Wroclaw, Wrocław, Poland; 9 Department of Pediatric Oncology, University of Zurich, Zurich, Switzerland; 10 St. Anna Kinderspital and St. Anna Kinderkrebsforschung e.V., Vienna, Austria; 11 Pediatric Oncology, Department of Women’s and Children’s Health, Uppsala University, Uppsala, Sweden; 12 Klinikum Stuttgart, Olgahospital, Pediatrics 5 (Oncology, Hematology, Immunology), Stuttgart, Germany; 13 Department of Pediatric Oncology, University of Tuebingen, Tubingen, Germany; 14 Hospital for Children and Adolescents, Goethe-University Frankfurt (Main), Frankfurt, Germany

**Keywords:** Synovial sarcoma, Soft-tissue sarcoma, Pediatric sarcoma, Adjuvant therapies, Chemotherapy, Radiotherapy

## Abstract

**Background:**

The benefit of adjuvant therapy in synovial sarcoma (SS) treatment is under debate. Long-term follow-up data are missing.

**Methods:**

SS patients treated in the consecutive trials CWS-81, CWS-86, CWS-91, CWS-96, CWS-2002-P, and the SoTiSaR-registry till 2013 were analyzed.

**Results:**

Median age of 185 patients was 13.9 years (0.1–56)—with median follow-up of 7.4 years for 163 survivors. Most tumors (76%) were located in extremities. Size was < 3 cm in 58 (31%), 3–5 cm in 59 (32%), 5–10 cm in 42 (23%), and > 10 cm in 13 (7%) (13 missing). In 84 (45%) tumors, first excision was complete (R0 corresponding to IRS-I-group) and in 101 (55%) marginal (R1 corresponding to IRS-II-group). In a subsequent surgical intervention during chemotherapy, R0-status was accomplished in 23 additional IRS-II-group patients with secondary surgery. Radiotherapy was administered to 135 (73%), thereof 62 with R0-status and 67 R1-status (6 missing information). Adjuvant chemotherapy was administered to all but six patients. 5-year event-free (EFS) and overall survival (OS) was 82.9% ± 5.7 (95%CI) and 92.5% ± 3.9. Local and metastatic relapse-free survival was 91.3% ± 4.3 and 92.3% ± 4.1 at 5 years, respectively. In the multivariate analysis, tumor size and no chemotherapy were independently associated with EFS. Size and site were associated with OS. In a detailed analysis of local and metastatic events, tumor size was associated with an independent risk for developing metastases. No independent factor for suffering local recurrence could be identified.

**Discussion:**

Omission of chemotherapy in a non-stratified way seems not justified. Size governs survival due to high linear association with risk of suffering metastatic recurrence in a granular classification.

**Supplementary Information:**

The online version contains supplementary material available at 10.1007/s00432-021-03614-6.

## Introduction

Soft-tissue sarcomas represent nearly 8% of childhood malignancies. Synovial sarcoma (SS) is the most common non-rhabdomyosarcoma soft-tissue sarcoma (Goldblum 2014; Pizzo and Poplack 2011), characterized by the specific translocation t(X;18). It typically affects the extremities of adolescents, as well as of young adults. The main age range is between 10 and 40 years. Pathological features are identical in all age groups (Goldblum 2014; Pizzo and Poplack 2011).

Whereas pediatric oncologists assumed that chemotherapy might play an important role, and administered adjuvant chemotherapy regardless of risk factors (Ferrari et al. 1999, 2004; Schmidt et al. 1991; Ladenstein et al. 1993; Pappo et al. 1994; Okcu et al. 2001, 2003), adult oncologists considered SS as a tumor with uncertain chemosensitivity (Frustaci et al. 2001; Sarcoma Meta-analysis Collaboration 1997; Brodsky et al. 1992; Bergh et al. 1999; Lewis et al. 2000; Spillane et al. 2000; Trassard et al. 2001). Pediatric trials reported very satisfactory results with up to 75–80% of survival rates (Ferrari et al. 1999, 2004; Schmidt et al. 1991; Ladenstein et al. 1993; Pappo et al. 1994; Okcu et al. 2001, 2003), better than those in adult series (Ferrari et al. 2004; Frustaci et al. 2001; Sarcoma Meta-analysis Collaboration 1997; Brodsky et al. 1992; Bergh et al. 1999; Lewis et al. 2000; Spillane et al. 2000; Trassard et al. 2001)—evolving the discussion if age per se is a risk factor (Hayes-Jordan et al. 2000) or if this is caused by the differing treatment approaches (Baldi et al. 2019; Tarkan et al. 2014; Italiano et al. 2009; Outani et al. 2019). Currently, treatment strategies converge towards more common strategies. However, especially for adolescents, still existing controversies cause discussions. Apart from systemic treatment, there is also disagreement about the required local aggressiveness. While, some reported resections with free margins to be crucial, especially pediatric series reported no differences in resections with positive margins (Orbach et al. 2011). Summarized, adjuvant therapies in grossly resected SS are still a matter of debates.

## Patients and methods

Patients treated 1980–2013 in the trials CWS-81 (Koscielniak et al. 1992), CWS-86 (Koscielniak et al. 1999), CWS-91 (Dantonello et al. 2009), CWS-96 (Modritz et al. 2005), CWS-2002-P (Koscielniak et al. 2013), and the registry CWS-SoTiSaR were eligible if (i) diagnosis was proven by central reference review (including molecular confirmation since 2000), (ii) no evidence of metastases existed, (iii) no previous treatment was performed, and (iv) the tumor was initially grossly resected.

All CWS-trials were prospective and approved by appropriate ethics committees. Written informed consent was obtained from patients, guardians/parents, or both (Dantonello et al. 2008, 2009). Clinical information, treatment data, and outcome were available for all. Some had been included in previous analysis (pathological slides were reviewed for the purposes of those studies) (Stegmaier et al. 2017; Scheer et al. 2016).

Disease was staged according to the clinical tumor-node-metastases (TNM) classification (Harmer et al. 1970) which confines T1-tumors to the organ/tissue of origin, while T2-lesions invade contiguous structures and regional node involvement as N0 or N1, based on histological or clinical/radiological assessments (Baldi et al. 2019). Originally developed for rhabdomyosarcoma, but extended to other chemotherapy-sensitive pediatric STS, the Intergroup Rhabdomyosarcoma Study (IRS) post-surgical grouping system (Maurer et al. 1988) categorizes patients based on the extent of residual tumor after first surgery: primary completely excised tumors with negative microscopic margins (R0) correspond to IRS-I; primary grossly resected tumors with microscopic residual disease (R1): IRS-II; macroscopic residual disease after incomplete resection or biopsy (R2): IRS-III; and metastases at onset: IRS-IV (Maurer et al. 1988).

Generally, a primary excision was attempted if complete and non-mutilating resection was considered feasible. Any re-surgery performed up to 4 weeks after biopsy/inadequate primary resection before any other treatment was defined as primary re-excision and considered in the IRS-system. The consecutive CWS-protocols recommended chemotherapy in all patients. The regimens were VACA (vincristine, actinomycin-D, cyclophosphamide, adriamycin) in CWS-81 (Koscielniak et al. 1992), and VAIA, which incorporates ifosfamide instead of cyclophosphamide, in CWS-86 (Koscielniak et al. 1999) and in all protocols including and following CWS-96 (Modritz et al. 2005; Koscielniak et al. 2013). The CWS-91-trial investigated therapy intensification with etoposide (EVAIA) (Dantonello et al. 2009). Since the CWS-96-protocol, the VAIA-regimen is used (Modritz et al. 2005; Koscielniak et al. 2013).

Radiotherapy was recommended for all except for IRS-I (primary R0) patients, where it was only recommended in CWS-86 and CWS-91. According to the respective CWS-protocol radiotherapy at doses of 32–54.4 Gy (when accelerated hyperfractionated) and 40–50 Gy if conventional fractionated dependent on response to chemotherapy and resection status was to be administered in analogy to recommendations for patients with rhabdomyosarcoma (Koscielniak et al. 1992, 1994, 1999; Dantonello et al. 2009; Modritz et al. 2005).

Best surgery was defined as the best surgical result at the end of treatment independent from the number of procedures. It was categorized as the presence of microscopic [R1] residual tumor or as resection with free margins [R0].

### Statistical methods

Statistics were calculated using SPSS® 24 (IBM SPSS, Armonk, NY, USA). Comparison of distribution was performed with the χ^2^-test. Event-free survival [EFS] and overall survival [OS] were calculated using the Kaplan–Meier estimator (Kaplan and Meyer 1958). For OS, time from diagnosis to death or last follow-up was calculated, for EFS time from diagnosis to first relapse/progression, death, or last follow-up. The local relapse-free survival [LRFS] was calculated from diagnosis to local (included combined) relapse. Metastases-free survival [MFS] was calculated from diagnosis to the onset of distant metastases. Patients who died of their tumor after distant failure, prior to local progression/relapse, were censored at the time of death in the analysis of LRFS. Confidence intervals [CI] for the Kaplan–Meier estimator were computed using Greenwoods Formula (Greenwood 1926) and are stated at the 95% level. For comparison of EFS, OS, LRFS, and MFS levels, the log-rank test was used. The Cox proportional hazard regression model was used to assess the effects of each potential prognostic variable on survival rates (Orbach et al. 2011). A stepwise variable selection procedure (combination of forward and backward selection techniques) was applied to the covariates with a p value of at least 0.05 in EFS, OS, LRFS, or MFS at the univariable analysis. Hazard ratios (HRs) with 95% CIs, calculated according to the Wald method, are reported for the evaluated variables.

## Results

### Characteristics

Among 330 patients with localized SS, 185 had undergone initial gross resection, thereof 84 with primary free margins IRS-I/(R0) and 101 with primary positive margins IRS-II/(R1). Gender distribution was balanced (Table 1). Median age was 13.9 years (first month of life–56.8). Localization of the primary tumor was: extremities 140 (76%), head–neck 10 (5%), shoulder or hip 20 (11%), and trunk 12 (8%). Local invasiveness (T2) was reported in 49 (26%) and size > 5 cm in 59 (32%). Six patients (3%) had nodal involvement.

**Table 1:** Univariate analysis of 185 IRS-I and IRS-II patients

	*N* (%)	5 yr EFS (95% CI)	*p* value	5 yr OS (95% CI)	*p* value	5 yr LRFS (95% CI)	*p* value	5 yr MFS (95% CI)	*p* value
Studies									
CWS 81	17 (9)	88.2 ± 15.3	*0.862*	87.8 ± 15.9	*0.819*	100%	*0.912*	93.8 ± 12.0	*0.843*
CWS-86	27 (15)	81.5 ± 14.7		92.6 ± 9.8		88.6 ± 12.2		88.6 ± 12.2	
CWS-91	18 (10)	76.6 ± 20.2		88.2 ± 15.3		87.7 ± 16.1		88.1 ± 15.5	
CWS-96	59 (32)	85.9 ± 9.0		92.5 ± 7.1		91.3 ± 7.3		92.4 ± 7.3	
CWS 2002P	39 (21)	83.8 ± 12.0		97.3 ± 5.3		91.9 ± 8.8		94.7 ± 7.3	
SoTiSaR	25 (14)	75.1 ± 20.6		91.8 ± 10.8		91.3 ± 11.6		95.7 ± 8.4	
Gender									
Female	94 (51)	87.9 ± 6.7	*0.295*	94.4 ± 4.7	*0.133*	94.3 ± 4.9	*0.335*	95.6 ± 4.3	*0.254*
Male	91 (49)	77.6 ± 9.0		90.4 ± 6.3		88.0 ± 7.1		88.7 ± 7.1	
Age (years)									
≤ 10	36 (19)	88.6 ± 10.6	*0.872*	94.3 ± 7.6	*0.926*	91.2 ± 9.6	*0.258*	96.4 ± 6.7	*0.053*
10–21	133 (72)	82.5 ± 6.7		92.8 ± 4.5		90.3 ± 5.3		93.5 ± 4.3	
≥ 21	16 (9)	73.3 ± 22.3		85.6 ± 18.6		100%		73.3 ± 22.3	
Site									
Extremities	140 (76)	84.1 ± 6.3	*0.502*	94.6 ± 3.9	***0.017***	92.1 ± 4.7	*0.799*	93.0 ± 4.3	*0.431*
Head–neck	10 (5)	90.0 ± 18.6		100%		100%		100%	
Shoulder or hip	20 (11)	85.0 ± 15.7		84.4 ± 16.3		90.0 ± 13.1		94.7 ± 10.0	
Trunk	15 (8)	66.7 ± 23.9		80.0 ± 20.2		80.0 ± 20.2		76.9 ± 22.9	
Size									
< 3 cm	58 (31)	89.8 ± 8.6	***0.011***	100%	** < *0.001***	89.8 ± 8.6	***0.037***	100%	***0.002***
3–5 cm	59 (32)	91.2 ± 7.3		98.3 ± 3.3		98.2 ± 3.5		94.5 ± 6.1	
5–10 cm	42 (23)	75.4 ± 13.3		84.9 ± 11.2		84.8 ± 11.2		89.2 ± 10.0	
> 10 cm	13 (7)	53.8 ± 27.0		69.2 ± 25.1		84.6 ± 10.0		65.8 ± 27.6	
No information	13 (7)								
Size (5 cm)									
≤ 5 cm	119 (64)	90.8 ± 5.5	***0.004***	99.2 ± 1.6	** < *0.001***	94.3 ± 4.5	***0.048***	97.2 ± 1.6	***0.005***
> 5 cm	59 (32)	70.6 ± 11.8		82.5 ± 9.8		85.5 ± 9.2		85.2 ± 9.6	
No information	7 (4)								
T-status									
T1	130 (70)	86.2 ± 6.1	*0.058*	95.2 ± 3.7	***0.040***	91.6 ± 4.9	*0.609*	94.0 ± 4.3	***0.032***
T2	49 (26)	73.1 ± 12.5		85.0 ± 10.4		89.4 ± 8.8		87.3 ± 9.6	
TX	6 (3)								
N-status									
N0	170 (92)	82.2 ± 5.9	*0.757*	91.8 ± 4.3	*0.857*	90.5 ± 4.5	*0.938*	91.6 ± 4.3	*0.725*
N1	6 (3)	100%		100%		100%		100%	
NX	9 (5)								
IRS									
IRS-I	84 (45)	79.1 ± 8.8	*0.119*	89.9 ± 6.7	*0.449*	89.6 ± 6.9	*0.459*	89.8 ± 6.7	*0.435*
IRS-II	101 (55)	86.3 ± 9.6		94.7 ± 4.5		92.9 ± 5.1		94.5 ± 4.7	
Chemotherapy									
No	6 (3)	22.2 ± 37.6	** < *0.001***	62.5 ± 41.5	***0.003***	44.4 ± 43.5	** < *0.001***	75.0 ± 42.5	***0.001***
VACA	30 (16)	83.1 ± 13.5		86.0 ± 12.7		92.7 ± 9.8		93.1 ± 9.2	
VAIA*	141 (76) 7 (4)	85.7 ± 5.88		95.4 ± 3.5		93.3 ± 4.3		93.1 ± 4.3	
No information									
Radiotherapy									
Yes	135 (73)	85.1 ± 6.3	*0.189*	95.3 ± 3.7	*0.086*	93.5 ± 4.3	*0.155*	92.1 ± 4.7	*0.572*
No	43 (23)	73.2 ± 13.7		82.2 ± 12.2		82.6 ± 11.8		91.3 ± 9.4	
No information	7 (4)								
Best surgery									
R0	107 (58)	81.7 ± 7.4	*0.548*	91.1 ± 5.5	*0.581*	90.9 ± 5.7	*0.988*	91.0 ± 5.7	*0.475*
R1	70 (38)	84.7 ± 8.8		93.8 ± 5.9		91.2 ± 6.7		95.1 ± 5.5	
No information	8 (4)								

Bold values indicate statistical significance

*Thereof 1 patient with EVAIA

### Treatment

Chemotherapy was given to all but six: 141 (76%) received the VAIA, 1 the EVAIA, and 30 (16%) the VACA regimen.

In the IRS-II/(R1) group, 23 of 101 patients underwent second surgical intervention only after the start of chemotherapy to obtain a tumor cell-free primary site. Therefore, in total, 107 (58%) achieved a R0-status as best surgical result at any time.

**Fig. 1:**
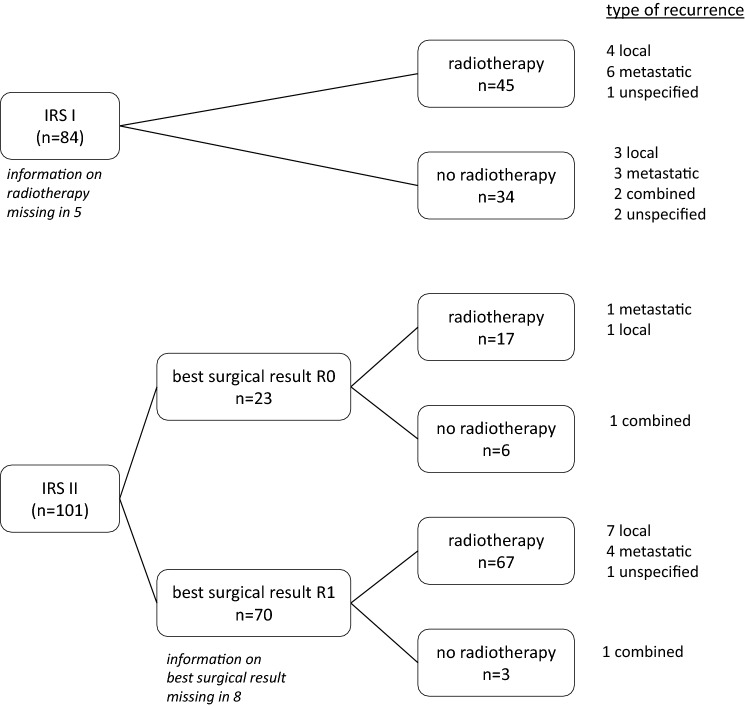
Pattern of relapse according to surgery and radiotherapy

Radiotherapy was administered to 135 (73%): 45 out of 84 (54%) IRS-I/(R0)-patients and 89 out of the 101 (88%) IRS-II/(R1) (detailed presentation in Fig. 1). The documented total dose ranged from 32 to 60 Gy, while the majority received < 50 Gy.

### Outcome

At last follow-up, 163/185 (88%) were alive. Median follow-up for survivors was 7.4 years (0.7–31.1). Twenty patients died of disease, two of treatment toxicity (according to the documentations of the participating centers: 1. “cardiogenic shock, cachexia” in first-line therapy while receiving the VACA regimen, 2. “liver failure, organ failure” in relapse chemotherapy).

3-, 5-, and 10-year EFS was 87.9% ± 4.7, 82.9% ± 5.7 and 75.2% ± 7.4. 3-, 5- and 10-year OS was 95.0% ± 3.1, 92.5% ± 3.92 and 84.6% ± 6.7, respectively.

### Pattern of relapse

39 patients developed recurrences, 16 local, 14 metastatic, 4 at local and distant sites combined. For 5 patients, type of relapse was not specified (Table 2). 3-, 5-, and 10-year LRFS and MFS were 93.9% ± 3.5, 91.3% ± 4.3, 86.9% ± 5.9 and 94.9% ± 3.3, 92.3% ± 4.1, 87.7% ± 6.1, respectively. Median time to local failure was 2.5 years. The latest local recurrences were documented at 7.7, 8.6, 11.5, and 16.0 years, respectively. Median time to distant failure without involvement of the primary region was 2.7 years. The latest occurrence of metastases was documented at 4.0, 5.8, 6.2, and 8.6 years.

**Table 2:** Failures and outcome according to IRS-group

	IRS-I	IRS-I (%)	IRS-II	IRS-II (%)
All patients	84	100	101	100
No relapse	62	74	84	83
Relapse	22	26	17	17
Local	8	10	8	8
Metastatic	9	11	5	5
Combined	2	2	2	2
Not specified	3	4	2	2
Total failures	22	26	17	17
Alive	72	86	91	90
Dead	12	14	10	10
Median follow-up for survivors, years (range)	7.6 (1.8–31.1)		7.1 (0.1–16.6)	

*CR* complete remission, *IRS* Intergroup Rhabdomyosarcoma Stage (IRS-I free margins, IRS-II positive margins)

The proportion of metastatic recurrences increased with larger tumor size. The rate of local relapses did not differ with size (Table 3).

**Table 3:** Type of relapse according to primary tumor size in 185 IRS-I and IRS-II group patients

Size of primary	< 3 cm (*n* = 58)			3–5 cm (*n* = 59)			5–10 cm (*n* = 42)			> 10 cm (*n* = 13)		
IRS stage	IRS-I (*n* = 24)	IRS-II (*n* = 34)	Total (*n* = 58)	IRS-I (*n* = 31)	IRS-II (*n* = 28)	Total (*n* = 59)	IRS-I (*n* = 21)	IRS-II (*n* = 21)	Total (*n* = 42)	IRS-I (*n* = 4)	IRS-II (*n* = 9)	Total (*n* = 13)
Best surgical result at any time		R0 (*n* = 10)			R0 (*n* = 7)			R0 (*n* = 1)			R0 (*n* = 3)	
Local relapse	4 (17%)	4 (12%)	**8 (29%)**	1 (3%)	1 (4%)	**2 (7%)**	3 (14%)	2 (10%)	**5 (24%)**	0	1 (11%)	**1 (11%)**
Metastatic relapse	1 (4%)	0	**1 (4%)**	2 (6%)	2 (7%)	**4 (13%)**	3 (14%)	1 (5%)	**4 (19%)**	2 (50%)	2 (22%)	**4 (72%)**
Combined relapse	0	0	**0**	0	0	**0**	1 (5%)	1 (5%)	**2 (10%)**	0	1 (11%)	**1 (11%)**
Unspecified relapse	0	0	**0**	1 (4%)	1 (4%)	**2 (8%)**	1 (5%)	0	**1 (5%)**	0	0	**0**

Bold letters indicate total values per size category

No detailed information on primary tumor size available in 13 patients

### Factors for survival

In the univariate analysis, factors associated with adverse EFS were large tumor size and no application of chemotherapy (Table 1, Fig. 1). Factors associated with adverse OS were tumor location at the head–neck, large tumor size, invasiveness (T2-status), and no application of chemotherapy. Survival was not associated with surgical margins.

In the Cox regression analysis, large tumor size and no application of chemotherapy were associated with adverse EFS. Large tumors and tumors localized at head–neck were associated with adverse OS (Table 4).

**Table 4:** Multivariate analysis of clinical and treatment variables for EFS, OS, LRFS and MFS

Variables	EFS Hazard ratio	CI (95%) lower	CI (95%) upper	*p* value	OS Hazard ratio	CI (95%) lower	CI (95%) upper	*p* value	LRFS Hazard ratio	CI (95%) lower	CI (95%) upper	*p* value	MFS Hazard ratio	CI (95%) lower	CI (95%) upper	*p* value
Age (years)																
≤ 10	1			*0.833*	1			*0.900*	1			*0.415*	1			*0.258*
10–21	1.144	0.381	3.433	*0.810*	0.812	0.188	3.497	*0.780*	0.447	0.136	1.469	*0.185*	1.050	0.184	5.993	*0.956*
≥ 21	1.624	0.320	8.249	*0.559*	1.231	0.096	15.769	*0.873*	0.000	0.000		*0.983*	3.552	0.408	30.946	*0.251*
Site																
Extremities	1			*0.148*	1			*0.078*	1			*0.825*	1			*0.252*
Head–neck	4.109	1.101	15.335	***0.035***	14.203	1.571	128.428	***0.018***	0.704	0.068	7.293	*0.769*	7.399	0.898	60.936	*0.063*
Shoulder–hip	1.206	0.342	4.253	*0.771*	3.781	0.865	16.522	*0.077*	1.407	0.289	6.854	*0.673*	0.821	0.098	6.873	*0.856*
Trunk	0.707	0.159	3.140	*0.649*	2.599	0.553	12.202	*0.226*	0.420	0.048	3.689	*0.434*	0.781	0.086	7.089	*0.826*
Size																
< 3 cm				***0.025***	1			***0.011***	1			*0.197*	1			***0.039***
3–5 cm	0.698	0.248	1.959	*0.494*	3.203	0.323	31.809	*0.320*	0.256	0.051	1.281	*0.097*	3.393	0.364	31.627	*0.283*
5–10 cm	2.065	0.717	5.947	*0.179*	17.719	1.740	180.444	***0.015***	1.497	0.451	4.969	*0.510*	9.285	0.883	97.669	*0.063*
> 10 cm	4.492	1.217	16.588	***0.024***	41.462	3.204	536.641	***0.004***	1.714	0.256	11.490	*0.579*	28.480	2.205	367.925	***0.010***
T-Status																
T1	1				1				1				1			
T2	1.242	0.541	2.852	*0.609*	0.988	0.316	3.091	*0.984*	1.448	0.460	4.555	*0.527*	1.084	0.319	3.686	*0.897*
Chemo																
VAIA*	1			***0.002***	1			*0.183*	1			*0.178*	1			*0.238*
VACA	1.836	0.731	4.613	*0.196*	3.142	0.852	11.587	*0.086*	1.687	0.502	5.673	*0.398*	1.477	0.361	6.042	*0.588*
No	12.903	2.764	60.226	***0.001***	2.664	0.262	27.097	*0.408*	6.378	0.665	61.200	*0.108*	7.766	0.686	87.927	*0.098*
Radiotherapy																
Yes	1				1				1				1			
No	1.211	0.472	3.106	*0.691*	2.328	0.688	7.877	*0.174*	1.959	0.588	6.527	*0.273*	0.556	0.098	3.160	*0.508*

Bold values indicate statistical significance

*Thereof 1 with EVAIA

### Factors for local and metastatic events

In the detailed evaluation of local and metastatic events, size and application of chemotherapy correlated with LRFS. Size, invasiveness (T-status) and chemotherapy correlated with MFS. In the Cox regression analysis, no independent factors for LRFS were identified. Size was associated with independent risk for MFS.

### Chemotherapy

When we analyzed patients treated with cyclophosphamide-based regimens (VACA) versus those treated with ifosfamide-based regimen (VAIA) versus those without chemotherapy, patients treated with the VAIA or the VACA scheme did not show different outcomes (5-year EFS and OS were 83.1% and 86.0% for VACA and 85.7% and 95.4% for VAIA, respectively), whereas 6 patients without chemotherapy did significantly worse (5-year EFS and OS were 22.2% and 62.5%; *p* < 0.001).

Among those six patients treated with local therapy alone 2 were reported to be in ongoing complete first remission at 12.4 years (primary tumor size < 3 cm) and 0.7 years (size missing) after first diagnosis. One suffered local relapse at 1.8 years (primary size 5–10 cm); he is reported alive in second complete remission 10.2 years after first diagnosis. Two suffered combined relapses at 0.3 years (primary tumor size 5–10 cm) and 0.5 years (size missing); both are dead of disease 0.5 and 2.8 years after diagnosis. One patient presented with metastatic relapse 1.5 years (primary tumor size 3–5 cm) after diagnosis and was reported alive at 3.8 years.

### Radiotherapy

Univariate analysis showed no significant correlation between radiotherapy and survival rates for the entire cohort.

The analysis of merely IRS-I/(R0)-patients revealed that 5-year EFS was 81.8 ± 11.4% for patients receiving radiotherapy and 72.2 ± 15.5% for those who did not (supplementary table 1). The application of radiotherapy was also not associated with local or metastatic relapse risk.

For IRS-II/(R1)-patients, EFS was 86.7 ± 3.7% for irradiated (*n* = 89) and 77.8 ± 13.9% for non-irradiated patients (*n* = 9), while 5-year OS was 96.3 ± 2.1% and 77.8 ± 13.9%, respectively. There was no significant association with local or metastatic recurrences (supplementary table 2).

In the subgroup of 23 IRS-II/(R1)-patients with secondary R0-resection while on chemotherapy, 6 did not receive radiotherapy. One of those 6 suffered combined relapse, whereas the 5 remaining were reported in ongoing first remission for 4.9 years (2.2–10.4). 17 did receive additional radiotherapy to the secondary R0-resection, and one suffered metastatic and one local relapse (Fig. 1, supplementary table 2).

**Fig. 2:**
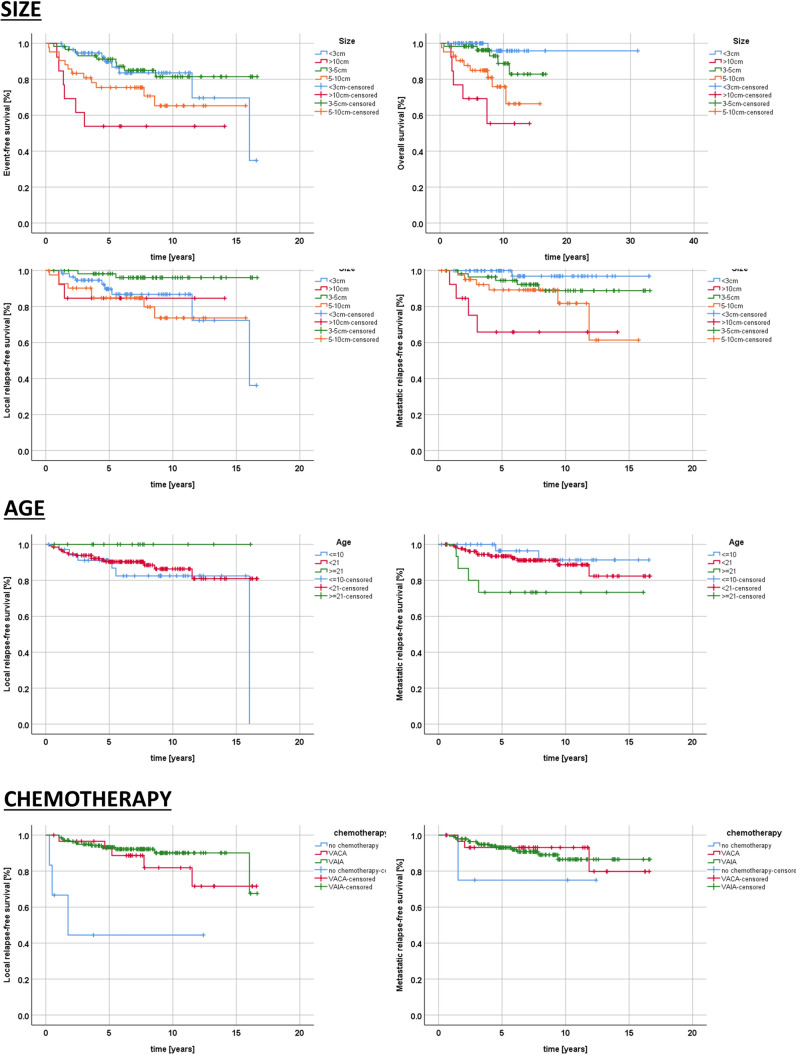
EFS, OS, LRFS and MFS according to tumor size. LRFS and MFS according to patients’ age. LRFS and MFS according to chemotherapy

### Small tumors

In the subgroup of those 56 patients with tumors < 5 cm and resection with negative margins IRS-I/(R0), 3-year-EFS was 94.6% ± 5.9. 5-year-EFS was 90.5% ± 8.0, 10-year-EFS 79.1% ± 12.9, and the 5-year-OS 98.2% ± 3.5. 52/56 received chemotherapy (38 VAIA, 14 VACA). 2 did not receive chemotherapy, while in 2 information was missing. With median follow-up of 7.5 years (0.6–31.1), 9/56 suffered relapse, thereof 5 local, 3 metastatic, 1 unspecified. 4/9 events occurred after 5.2 years.

In the subgroup of 63 patients with tumors < 5 cm and resection with positive margins IRS-II/(R1), 3-year-EFS was 93.3 ± 6.3, 5-year-EFS 91.3 ± 7.3, 10-year-EFS 86.1 ± 9.8, and the 5-year-OS was 100%. This does not differ to the results of the IRS-I/(R0)-patients. All 63 patients received chemotherapy (53 VAIA, 9 VACA) with 1 missing information. 8 suffered relapse with a median follow-up of 6.8 years (1.2–16.6), thereof 5 local, 2 metastatic, 1 unspecified. Median time to relapse was 3.2 years (1.3–16.0).

### Secondary malignancies and long-term toxicities due to radiotherapy and chemotherapy

Secondary malignancy was documented in 4 patients: [(1) benign ganglioneuroma and enchondroma of the fibula, (2) melanoma in situ, left foot, (3) embryonal carcinoma of the testis, and (4) basal cell carcinoma]. All patients achieved a remission of their secondary tumor.

Information on late effects was available for 142 patients. Thereof 72 patients (51%) did not suffer any late effect. Among those 70 other patients, mainly late sequelae in the extremities was generally documented by the participating centers. Among the 70 patients in the whole, 12 were documented with renal dysfunction, 8 with neuropathy, and 2 with cardiomyopathy. 57 of those 70 patients were irradiated. In 10 irradiated patients, specifically loss of function of their extremity was documented and in 3 other irradiated patients leg length differences or growth problem of the limb.

## Discussion

Whereas pediatric and adult studies mostly agree on factors influencing SS outcome (Ferrari et al. 1999, 2004; Schmidt et al. 1991; Ladenstein et al. 1993; Pappo et al. 1994; Okcu et al. 2001, 2003; Brodsky et al. 1992; Bergh et al. 1999; Lewis et al. 2000; Spillane et al. 2000; Trassard et al. 2001; Koscielniak et al. 1992, 1999; Dantonello et al. 2009), there is no consensus on how patients should be classified to receive adjuvant therapy. In particularly, those factors essential for risk stratification in relation to chemotherapy have not yet been defined uniformly across age groups. All 185 patients analyzed here are treated in prospective trials with median follow-up of more than 7 years for survivors. We can conclude that children, adolescents, and adults with grossly resected SS treated according to CWS recommendations have an excellent prognosis with an expected 5-year and 10-year OS of 93% and 85%, respectively. Regarding first relapse, patients had a local recurrence rate of 18%, a distant metastases rate of 16%, and a combined relapse rate of both local and distant lesions of 4%. Despite this excellent outcome, patients with large and very large tumors and those without chemotherapy are at independent risk for adverse events. The evaluation of distinct event types reveals that the risk of suffering metastatic recurrence is independently and moreover linearly associated with large tumors in a granular size classification. No independent factor for local recurrence was identified.

All except for six patients received chemotherapy. This small unselected subgroup has refused chemotherapy. Multivariate analysis proves independent impact of chemotherapy. However, results might be compromised by a low number in the non-chemotherapy group and can only be interpreted in consideration with the literature. In a prior attempt, the CWS and Italian IGC have reviewed the data of grossly resected SS in 2006. The study identified a subset of low-risk patients (IRS-I, < 5 cm), for which the omission of adjuvant chemotherapy might be recommended (Brecht et al. 2006). Consequently, according to the EpSSG-recommendations 2005, those patients were treated with surgery only. Patients with tumors < 5 cm and/or resection with positive margins were recommended to have chemotherapy and radiotherapy (Ferrari et al. 2015). In the COG ARST0332 trial (NCT00346164), for newly diagnosed non-rhabdomyosarcoma soft-tissue sarcoma, starting in 2007, patients with low-grade tumor with either negative or positive microscopic margins or high-grade tumor ≤ 5 cm with negative margins had not received adjuvant or further therapy. In 2017, Ferrari et al. reported results of 3-year EFS of 90% in 60 patients with median follow-up of 5.2 years. All eight events were local recurrences. All were effectively salvaged. The authors consequently conclude that adequately resected SS < 5 cm, regardless of grade can be safely treated with a surgery only approach.

In our subgroup of those 56 patients with tumors < 5 cm resected with negative margins (IRS-I/R0), 3-year EFS was 94.6% ± 5.9. This does not differ from the results without chemotherapy. In our series, 9/56 suffered relapse, thereof 5 local, 3 metastatic, 1 unspecified with median follow-up of 7.5 years. 4/9 events occurred after 5.2 years. Median follow-up for the Ferrari series is merely 5.2 years. Long-term results are therefore highly anticipated. International cooperation with biological accompanying investigation would be needed to finally resolve this question.

In our subgroup of 63 patients with tumors < 5 cm and resection with positive margins (IRS-II/R1), 3-year-EFS was 93.3% ± 6.3. This does not differ from the IRS-I/(R0)-group. There is neither a difference between IRS-I/(R0) and IRS-II/(R1)-group, nor between best surgeries obtained in a second intervention during chemotherapy. However, patients with positive margins mostly received irradiation. Nevertheless, it should be underlined that the risk of metastatic relapse does not differ. Obviously, distant spread occurs before resection. Therefore, omission of chemotherapy might be an approach worth discussing in grossly resected SS < 5 cm regardless of resection margins.

There are very few evaluations that deal with the question of which systemic anti-cancer drugs specific to SS improve outcome best (Baldi et al. 2019; Riedel et al. 2018). Knowledge of effective substances mainly derives from retrospective analyses and basket trials. The patients in our series received the VACA (vincristine, adriamycin, cyclophosphamide, actinomycin-D) or the VAIA (vincristine, adriamycin, ifosfamide, actinomycin-D) regimen. In the course of history, the combination VAIA has prevailed. In the present analysis, there was no significant difference between VACA and VAIA with a slightly better outcome with VAIA. Therefore, in patients who do not tolerate ifosfamide, the use of cyclophosphamide might not compromise outcome. Interestingly, the metastasis relapse-free survival of patients >  = 21 years is worse than that of patients < 21 years. Even though the indication for chemotherapy in adult synovial sarcoma patients is not standardized, the chemotherapy combination commonly used consists of doxorubicin/ ifosfamide. However, in this series, all patients were treated according to the respective pediatric protocol. The nonetheless poorer metastasis-free survival of patients >  = 21 years raises the question of biological differences in different age groups.

CWS-protocols recommend radiotherapy for all SS patients except for IRS-I/(R0), where it was only recommended in CWS-86 and CWS-91. There is no clear evidence of the role of radiotherapy in IRS-I/(R0). A favorable trend with no statistically significant difference was shown (Ferrari et al. 2004). In the CWS-Italian co-analysis (Brecht et al. 2006), no benefit was observed, irrespective of tumor size. Consequently, no radiotherapy for IRS-I/(R0)-patients is recommended regardless of size and T-status in the CWS recommendations.

In patients with initial complete macroscopic resection with positive margins (IRS-II/R1), the indication for radiotherapy is a matter of debate. Generally, it is considered as indicated. In the common analysis (Brecht et al. 2006), treatment results for IRS-II/(R1) patients were comparable to those in IRS-I/(R0). Nearly all IRS-II/(R1) patients received radiotherapy. Nevertheless, data from Orbach et al. showed similar outcome regardless of irradiation in a subset of 27 IRS-II/(R1) patients, thus suggesting that radiotherapy may not be necessary after microscopic incomplete surgery (Orbach et al. 2011). Interestingly, in our IRS-II/(R1) patients, the administration of radiotherapy was also not associated with improved survival or with reduced risk of local recurrence. In a closer look at the subgroup of those with R1-resection as best surgical result at the end of treatment and treated without radiotherapy, relapse rate did not differ from the subgroup of R0 resected SS and of those treated with radiotherapy (Fig. 1, supplementary table 2).

Interestingly, the degree of surgery also lacked its prognostic role—unlike others reported (Ferrari et al. 2004; Ladenstein et al. 1993; Pappo et al. 1994; Okcu et al. 2003; Harmer et al. 1970). The reasons that may partially explain differences might probably relate, at least in part, to the difficulty of a precise definition of IRS-II and R1 due to adequate surgical approach and adequate surgical margins. However, chemotherapy affects not only the potential spread distant micro-metastases, but also the primary or tumor cells that might have remained in the primary tumor area after gross resection. In this way, chemotherapy can also be considered as local therapy. It is a generally accepted fact that prognostic factors should not be interpreted apart from the particular study population and the therapeutic context. Beyond that, the aggressiveness of local therapy cannot be assessed correctly when considered independently of systemic therapies. To put this in more general terms, chemotherapy might reduce the required aggressiveness. Differences in the applied systemic therapies might explain the contradictory or inconsistent results (Tarkan et al. 2014; Yaser et al. 2014; Salcedo-Hernandez et al. 2013; Vining et al. 2017). However, in clinical use, this interpretation would be important in complicated local situations.

Unfortunately, grading was not available for many of the patients included during the long recruitment period. In former times, synovial sarcoma was generally considered as a high-grade tumor (Ferrari et al. 2015), whereas nowadays, tumor grade is considered to be a predictive factor (Bianchi et al. 2017; Guillou et al. 2004). Nevertheless, our group could not provide evidence for this (Stegmaier et al. 2017).

The consecutive CWS studies had general follow-up recommendations. These were not sarcoma subtype specific. There were only slight differences between the different studies. Regular routine examinations were recommended for 5 years after completion of therapy. Summarized, in the first year, regular cross-sectional imaging of the primary tumor region, preferably MRI, was recommended at 4-month intervals. In the following 2 years, at intervals of 6 months. After 3 years, intervals of 6–12 months were recommended. Chest-X-ray or CT thorax (at least every 6 months), ultrasound abdomen/pelvis (at least every 6 months), and a bone scan (risk-adapted, once a year) were recommended in the first year. Chest X-ray was recommended at 6-month intervals in the second year, followed by annual checks until 5 years after completion of therapy. From the 6th year onwards, controls by sonography/cross-section imaging of the primary tumor region and lung imagings were recommended with frequency at the discretion of the responsible physician. Interestingly, despite the known heterogeneity of soft-tissue sarcoma and in particularly of the group of NRSTS, there are no detailed subtype-specific follow-up recommendations. In this series of grossly resected SS, median time to local failure was 2.5 years. Median time to distant failure without involvement of the primary region was 2.7 years. This result might suggest regular imaging follow-up examinations at closer intervals (e.g., 4 months) until 3 years after the end of therapy, and then an extension of the intervals. The latest local recurrences were documented at 11.5 and 16.0 years and the latest occurrence of metastases at 6.2 and 8.6 years, respectively. Annual control examinations until ~ 10 years after the end of therapy might seem reasonable. The possibility of a very late relapse should be known by the treating physicians. Most relapses are, however, detected due to clinical signs and symptoms and the patients should be educated to contact their oncologist immediately in case of unclear symptoms. It seems reasonable to incorporate the identified risk factors in the follow-up recommendations, e.g., patients with tumors < 5 cm have a significantly lower risk of suffering metastatic recurrence (whereas 72% of patients with a SS > 10 cm suffer metastatic relapse, Table 3). In the future, it will be important to also incorporate the constantly growing knowledge of tumor biology or rather biological risk factors into the recommendations for follow-up care.

In summary, to our knowledge, this analysis represents the largest series of grossly resected SS treated in prospective risk-adapted trials so far. It adds interesting data that could be helpful in treatment decisions:

1. Tumor size governs survival in grossly resected SS regardless of all other factors. Moreover, tumor size is linearly associated with the risk of suffering metastatic relapse. Therefore, a granular size classification seems reasonable.
2. A non-risk stratified omission of chemotherapy results in a significant deterioration in outcome.
3. In those patients with tumors < 5 cm resected with negative margins, survival is not superior with chemotherapy. Though long-term survival data of those treated without chemotherapy are highly anticipated, omission of chemotherapy seems justified.
4. The survival of patients with tumors < 5 cm resected with positive margins does not differ from those with negative margins.
5. Moreover, no independent factor for suffering local recurrence could be identified.
6. This series is in contrast to many other SS series that identify free margins as crucial factor. However, this is a series where almost all patients have received chemotherapy. The required aggressiveness of local therapy cannot be assessed independently of the systemic therapy administered.

Summarized, patients with grossly resected SS treated according to CWS recommendations have an excellent prognosis. A subgroup probably does not require chemotherapy. Whether size and surgery can serve as criteria for the omission of chemotherapy needs to be tested in prospective preclinical and clinical studies. Biology signature may predict the outcome and may be used for patients' stratification to better identify patients more appropriate to receive systemic therapy.

## Supplementary Information

Below is the link to the electronic supplementary material.

Supplementary file1 (DOCX 35 kb)

Supplementary file2 (DOCX 33 kb)
