# Supplementary material for: The effect of adjuvant therapies on long-term outcome for primary resected synovial sarcoma in a series of mainly children and adolescents
Source: J Cancer Res Clin Oncol. 2021 Jul 17;147(12):3735–47. doi: 10.1007/s00432-021-03614-6 (PMC8557198; doi:10.1007/s00432-021-03614-6)
Supplement: Supplementary file 1 — Supplementary file1 (DOCX 35 kb) [file 432_2021_3614_MOESM1_ESM.docx]

Table 1. Univariate analysis of 84 included IRSI-patients (primary complete resection with free margins)

|  | ***N* (%)** | **5yr EFS**  **(95% CI)** | ***p* value** | **5yr OS**  **(95% CI)** | ***p* value** | **5yr LRFS**  **(95% CI)** | ***p* value** | **5yr MFS**  **(95% CI)** | ***p* value** |
| --- | --- | --- | --- | --- | --- | --- | --- | --- | --- |
| **All patients** | 84 (100) | 79.1±8.8 |  | 89.9±6.7 |  | 89.6±6.9 |  | 89.8±6.7 |  |
| **Studies**  CWS 81  CWS 86  CWS 91  CWS 96  CWS 2002P  SoTiSaR | 10 (12)  15 (18)  11 (13)  22 (26)  15 (18)  11 (13) | 80.0±24.7  80.0±24.7  80.8±23.9  81.8±16.1  77.1±22.9  72.7±26.3 | *0.851* | 78.8±26.3  93.3±12.5  90.9±17.1  90.7±12.3  100±0  81.8±22.7 | *0.692* | 100±0  85.7±18.4  88.9±20.6  90.9±12.0  84.4±20.0  90.0±18.6 | *0.975* | 88.9±20.6  86.7±17.2  90.9±17.1  85.6±15.1  100±0  90.0±18.6 | *0.633* |
| **Gender**  female  male | 43 (51)  41 (49) | 80.8±12.0  77.3±13.1 | *0.592* | 92.8±7.8  87.2±10.6 | *0.484* | 92.3±8.4  86.6±11.0 | *0.660* | 92.8±7.8  86.8±10.8 | *0.895* |
| **Age [years]**  ≤10  10-21  ≥21 | 15 (18)  60 (71)  9 (11) | 86.7±17.2  80.8±10.2  55.6±32.5 | *0.310* | 93.3±12.5  91.2±7.4  74.1±31.6 | *0.599* | 92.9±13.5  87.3±8.8  100±0 | *0.533* | 92.3±14.5  94.7±5.9  55.6±32.5 | ***0.003*** |
| **Site**  Extremities  Head-neck  Shoulder-hip  Trunk | 64 (76)  2 (2)  9 (11)  9 (11) | 77.5±10.4  100±0  100±0  66.7±30.8 | *0.684* | 90.2±7.4  100±0  100±0  77.8±27.2 | *0.181* | 89.6±7.8  100±0  100±0  77.8±27.2 | *0.628* | 90.1±7.6  100±0  100±0  75.0±30.0 | *0.219* |
| **Size**  <3cm  3-5cm  5-10cm  >10cm  no information | 24 (29)  31 (37)  21 (25)  4 (5)  4 (5) | 90.4±12.7  90.2±10.6  65.6±20.8  50.0±49.0 | *0.110* | 100±0  96.8±6.3  79.8±17.8  75.0±42.5 | *0.181* | 90.4±12.7  96.6±6.7  78.8±18.6  100±0 | *0.117* | 100±0  92.9±9.4  84.4±16.3  50.0±49.0 | ***0.006*** |
| **Size (5cm)**  <=5cm  >5cm  no information | 56 (67)  25 (30)  3 (4) | 90.5±8.0  63.1±19.2 | ***0.022*** | 98.2±3.5  79.2±16.3 | ***0.033*** | 93.9±6.7  81.8±16.3 | *0.112* | 96.1±5.3  82.9±15.3 | ***0.035*** |
| **T-Status**  T1  T2  TX | 63 (75)  20 (24)  1 (1) | 83.7±9.2  64.6±21.2 | *0.126* | 93.6±6.1  79.2±18.2 | *0.315* | 89.5±8.0  89.1±14.3 | *0.813* | 93.1±6.6  79.4±18.0 | ***0.055*** |
| **N-Status**  N0  N1  NX | 81 (96)  0 (0)  3 (4) | 78.3±9.2 |  | 89.5±6.9 |  | 89.1±3.7 |  | 89.4±6.9 |  |
| **Chemotherapy**  No  VACA  VAIA  no information | 4 (5)  18 (21)  56 (67)  0 (0) | 25.0±42.5  77.4±19.6  85.1±9.6 | ***0.004*** | 75.0±42.5  82.6±17.8  94.3±6.3 | *0.583* | 50.0±49.0  93.3±12.5  92.3±7.3 | ***0.008*** | 75.0±42.5  88.2±15.3  92.4±7.3 | *0.508* |
| **Radiotherapy**  yes  no  no information | 45 (54)  34 (40)  5 (6) | 81.8±11.4  72.2±15.5 | *0.706* | 93.2±7.4  83.6±13.3 | *0.461* | 92.4±8.2  84.1±12.9 | *0.666* | 88.6±9.4  89.5±11.4 | *0.489* |
| **Best surgery**  R0  no information | 84 (100) | 79.1±8.8 |  | 89.9±6.7 |  | 89.6±6.9 |  | 89.8±6.7 |  |
|  | **Median** | **EFS (range)**  6.6 (0.2-16.1) | | **OS (range)**  7.4 (0.2-31.1) | | **LRFS (range)**  6.7 (0.2-16.1) | | **MFS (range)**  7.1 (0.2-16.1) | |

Bold values indicate statistical significance
