# Supplementary material for: The effect of adjuvant therapies on long-term outcome for primary resected synovial sarcoma in a series of mainly children and adolescents
Source: J Cancer Res Clin Oncol. 2021 Jul 17;147(12):3735–47. doi: 10.1007/s00432-021-03614-6 (PMC8557198; doi:10.1007/s00432-021-03614-6)
Supplement: Supplementary file 2 — Supplementary file2 (DOCX 33 kb) [file 432_2021_3614_MOESM2_ESM.docx]

Table 1. Univariate analysis of 101 included IRS II patients (primary complete resection with positive margins)

|  | ***N* (%)** | **5yr EFS**  **(95% CI)** | ***p* value** | **5yr OS**  **(95% CI)** | ***p* value** | **5yr LRFS**  **(95% CI)** | ***p* value** | **5yr MFS**  **(95% CI)** | ***p* value** |
| --- | --- | --- | --- | --- | --- | --- | --- | --- | --- |
| **All patients** | 101 (100) | 86.3±6.9 |  | 94.7±4.5 |  | 92.9±5.1 |  | 94.5±4.7 |  |
| **Studies**  CWS 81  CWS 86  CWS 91  CWS 96  CWS 2002P  SoTiSaR | 7 (7)  12 (12)  7 (7)  37 (37)  24 (24)  14 (14) | 100±0  83.3±10.8  68.6±18.6  88.9±5.3  87.3±6.9  73.3±17.6 | *0.611* | 100±0  91.7±0  83.3±15.2  94.1±4.1  95.7±4.3  100±0 | *0.539* | 100±0  91.7±15.7  85.7±25.9  91.1±9.0  95.8±8.0  91.7±15.7 | *0.517* | 100±0  90.9±17.1  80.0±35.1  97.1±5.5  91.3±11.6  100±0 | *0.821* |
| **Gender**  female  male | 51 (50)  50 (50) | 94.0±3.3  77.9±6.2 | ***0.037*** | 95.8±2.9  77.9±6.2 | *0.179* | 96.0±5.5  89.7±8.6 | *0.380* | 98.0±3.9  90.4±9.0 | *0.103* |
| **Age [years]**  ≤10  10-21  ≥21 | 21 (21)  73 (72)  7 (7) | 90.2±6.6  84.0±4.4  100±0 | *0.493* | 94.7±5.1  94.2±2.8  100±0 | *0.643* | 90.2±12.9  93.0±5.9  100±0 | *0.241* | 100±0  92.4±6.5  100±0 | *0.547* |
| **Site**  Extremities  Head-neck  Shoulder-hip  Trunk | 76 (75)  8 (8)  11 (11)  6 (6) | 90.2±3.5  87.5±11.7  72.7±13.4  66.7±19.2 | *0.120* | 98.6±1.4  100±0  71.6±1.4  83.3±15.2 | ***0.004*** | 94.5±5.3  100±0  81.8±22.7  83.3±29.8 | *0.441* | 95.7±4.7  100±0  90.0±18.6  80.0±35.1 | *0.751* |
| **Size**  <3cm  3-5cm  5-10cm  >10cm  no information | 34 (34)  28 (28)  21 (21)  9 (9)  9 (9) | 90.6±5.2  92.4±5.1  85.2±7.9  55.6±16.6 | *0.056* | 100±0  100±0  90.2±6.6  66.7±15.7 | ***0.002*** | 90.6±10.2  100±0  90.2±12.5  77.8±27.2 | *0.207* | 100±0  96.3±7.1  94.1±11.2  74.1±31.6 | *0.105* |
| **Size (5cm)**  <=5cm  >5cm  no information | 63 (62)  34 (34)  4 (4) | 91.3±3.7  76.1±7.4 | *0.053* | 100±0  85.0±6.2 | ***0.001*** | 100±0  85.0±6.2 | *0.164* | 100±0  85.0±6.2 | *0.068* |
| **T-Status**  T1  T2  TX | 66 (65)  29 (29)  6 (6) | 88.7±4.1  79.2±7.6 | *0.163* | 96.8±2.2  89.5±5.7 | ***0.048*** | 93.8±6.0  89.7±11.2 | *0.554* | 94.8±5.9  92.8±9.6 | *0.221* |
| **N-Status**  N0  N1  NX | 88 (87)  6 (6)  7 (7) | 85.8±3.8  100±0 | *0.460* | 93.9±2.6  100±0 | *0.790* | 91.9±5.7  100±0 | *0.815* | 93.7±5.3  100±0 | *0.527* |
| **Chemotherapy**  EVAIA  No  VACA  VAIA  no information | 1 (2)  2 (2)  12 (12)  84 (83)  2 (2) | 0  0  91.7±8.0  87.3±3.8 | ***<0.001*** | 0  50.0±35.4  90.9±8.7  96.1±2.2 | ***<0.001*** | 0  50.0±69.4  91.7±15.7  93.9±5.3 | ***<0.001*** | 0  0  100±0  94.8±4.9 | ***-*** |
| **Radiotherapy**  yes  no  no information | 89 (88)  9 (9)  2 (2) | 86.7±3.7  77.8±13.9 | *0.475* | 96.3±2.1  77.8±13.9 | *0.107* | 94.2±4.9  77.8±27.2 | *0.102* | 93.8±5.3  100±0 | *0.438* |
| **Best surgery**  R0  R1  no information | 23 (23)  70 (69) | 91.1±6.0  84.7±4.5 | *0.381* | 95.7±4.3  93.8±3.0 | *0.916* | 95.7±8.4  91.2±6.7 | *0.525* | 95.2±9.0  95.1±5.5 | *0.989* |
|  | **Median** | **EFS (range)**  6.3 (0.3-16.4) | | **OS (range)**  7.1 (0.5-16.4) | | **LRFS (range)**  6.7 (0.3-16.4) | | **MFS (range)**  6.5 (0.5-16.4) | |

Bold values indicate statistical significance
